# Some Biological Consequences of the Inhibition of Na,K-ATPase by Translationally Controlled Tumor Protein (TCTP)

**DOI:** 10.3390/ijms19061657

**Published:** 2018-06-04

**Authors:** Jiwon Jung, Seonhyung Ryu, In A Ki, Hyun Ae Woo, Kyunglim Lee

**Affiliations:** Graduate School of Pharmaceutical Sciences, College of Pharmacy, Ewha Womans University, Seoul 120-750, Korea; major87@hanmail.net (J.J.); shryu8723@gmail.com (S.R.); inainakee@gmail.com (I.A.K.)

**Keywords:** autophagy, hypertension, Na,K-ATPase, translationally controlled tumor protein, tumorigenesis

## Abstract

Na,K-ATPase is an ionic pump that regulates the osmotic equilibrium and membrane potential of cells and also functions as a signal transducer. The interaction of Na,K-ATPase with translationally controlled tumor protein (TCTP) results, among others, in the inhibition of the former’s pump activity and in the initiation of manifold biological and pathological phenomena. These phenomena include hypertension and cataract development in TCTP-overexpressing transgenic mice, as well as the induction of tumorigenesis signaling pathways and the activation of Src that ultimately leads to cell proliferation and migration. This review attempts to collate the biological effects of Na,K-ATPase and TCTP interaction and suggests that this interaction has the potential to serve as a possible therapeutic target for selected diseases.

## 1. Introduction

Na,K-ATPase is a P-Type ATPase that acts as an ionic pump that helps to maintain osmotic equilibrium and membrane potential in cells. Na,K-ATPase has been the subject of numerous studies since its discovery as an ion pump [1]. The ionic and electrochemical gradients maintained by Na,K-ATPase play critical roles in numerous physiological processes, such as electrical excitability, ion reabsorption in kidney, regulation of cell volume, uptake of nutrients into the cell, and regulation of cardiac glycoside (CG)-mediated signaling pathways [2,3,4].

Na,K-ATPase interacts with translationally controlled tumor protein (TCTP), also known as TPT1, P23, fortilin, and histamine-releasing factor, a highly conserved protein, ubiquitous in multicellular organism [5], and is itself involved in the regulation of many fundamental processes, such as cell proliferation, apoptosis, autophagy, pluripotency, progression of the cell cycle, and cytokine-like activity [6] to promote manifold biological and pathological phenomena. This review attempts to present current information on these biological phenomena.

## 2. Na,K-ATPase, TCTP, and Their Interaction

### 2.1. Na,K-ATPase

Na,K-ATPase is a multi-subunit plasma membrane protein of P-type ATPases, which transport cations across the cell membrane. Using energy from ATP, the conformational transition of the α subunit couples the processes of phosphorylation and extrusion of three Na^+^ ions with the uptake of two K^+^ ions [7]. Na,K-ATPase plays an essential role in maintaining an electrochemical gradient across the cell membrane. The established ionic and electrochemical gradient plays critical roles in numerous physiological processes, such as electrical excitability, ion reabsorption in kidney, regulation of cell volume and osmotic pressure, and uptake of nutrients into the cell [8].

Na,K-ATPase consists of a catalytic α subunit and regulatory β and γ subunits [9]. Its 110 kDa α subunit is a binding site for ATP, cations, and cardiac glycosides and is composed of 10 transmembrane (TM) domains and three major cytoplasmic domains [10]. The Na,K-ATPase α subunit has four isoforms, which are distributed differently in different tissues [11]. The α1 isoform is found in all cells, mainly acting as a “housekeeping” isoform. The α2 isoforms are present in muscle, heart, glial cells, and adipocytes. The α3 isoforms are abundant in nervous tissues and skeletal muscle. The α4 subunits are distributed in the testis [12]. The *N*-glycosylated 55 kDa β subunit is composed of one transmembrane (TM) helix and acts as a chaperone protein in association with the Na,K-ATPase α subunit, which traffics the αβ heterodimer to the plasma membrane. The β subunit influences K^+^ ion binding affinity to Na,K-ATPase and plays an essential role in cell polarity [13,14]. The β isoforms are distributed tissue-dependently. β1 is ubiquitously expressed in all tissues. β2 was originally found as an adhesion molecule on glial cells mediating neuron and glia interactions and known to promote neurite outgrowth. β2 is also found in neurons, pineal gland, and skeletal muscle. β3 is primarily distributed in the testis, but also found in glial cells, liver, lung, and kidney. The α1β1 isoform is the main Na,K-ATPase isozyme, which provides the driving force for Na^+^ reabsorption. Diverse α and β isozyme combinations are present depending on the tissues and cell types [12]. The γ-subunit, often known as FXYD2 protein, is mainly expressed in the kidney. It interacts with Na,K-ATPase αβ heterodimers in a tissue-specific manner and modulates the activity of Na,K-ATPase by regulating ion channel activity [15].

Since Na,K-ATPase is important for maintaining various cellular functions, its inhibition could result in diverse pathologic states. Inhibition of Na,K-ATPase causes high intracellular Na^+^ ion levels and subsequent increases in intracellular Ca^2+^ ion through the Na^+^/Ca^2+^ exchanger [16]. Cardiac glycosides such as ouabain inhibit Na,K-ATPase and elevate the intracellular Ca^2+^ ion level, causing hypertension, cataracts, diabetes, and several other pathological events [17,18,19]. Recently, the activation of several signaling pathways by low-dose ouabain was identified without alteration in intracellular ionic concentration [8]. Inhibition of Na,K-ATPase by ouabain induced the activation of epidermal growth factor receptor (EGFR) in A7r5 and LLC-PK1 cells, leading to the identification of Src kinase as a key player in Na,K-ATPase signal transduction [20]. Several signaling pathways, such as Ras–Raf–MEK–MAPK, phospholipase-γ (PLC-γ), and phosphatidylinositide 3-kinase (PI3K) pathways have been identified as downstream signaling pathways of ouabain-induced Src kinase activation. These activated signaling pathways are involved in modulating gene expression related to cell growth, survival, and motility [21,22,23]. The diverse functions of Na,K-ATPase as a signal transducer as well as an ion transporter are still currently being investigated.

### 2.2. Translationally Controlled Tumor Protein (TCTP)

TCTP is a ubiquitous multifunctional protein first discovered in mouse sarcoma ascites cells [24]. TCTP has been implicated in many biological processes, such as cell growth [25], microtubule stabilization [26], allergic reaction [27], tumor reversion [28], and tumorigenesis [29]. One of its important function is its role in tumorigenesis. TCTP level in tumors is higher than in the corresponding normal tissues [30], and TCTP downregulation induced tumor reversion in diverse cancer cell lines [28]. TCTP has been reported to promote cell migration and invasion by inducing epithelial to mesenchymal transition (EMT) in LLC-PK1 cells [31]. Another important function of TCTP is its extracellular role as a histamine-releasing factor [27] which acquires cytokine-like function by dimerization [32]. Also, its importance in cell survival was discovered in TCTP-knockout mice, in which it was embryonic lethal at 6.5 day as a consequence of increased cell death [33].

TCTP interaction with different binding partners produces diverse biological effects (Table 1). TCTP interacts with Bcl-xL [34] and Mcl-1 [35] and increases their stability, thereby providing anti-apoptotic effects. By interacting with heat shock protein 27 (Hsp27), a protein highly overexpressed in castration-resistance prostate cancer (CRPC), TCTP mediates Hsp27-induced cytoprotection in CRPC cells [36]. TCTP also interacts with p53 and inhibits p53-induced transcriptional activation of Bax [37], and p53 and TCTP are known to act as reciprocal regulators [38]. TCTP also interacts with MDM2 and inhibits ubiquitination of MDM2, inducing p53 degradation [39]. Polo-like kinase interacts and phosphorylates TCTP, thereby decreasing the microtubule-stabilizing effect of TCTP [26]. It has been shown that TCTP interaction with Na,K-ATPase occurs via the third cytoplasmic domain of Na,K-ATPase α subunit and inhibits the latter’s pumping activity, leading to a pathological phenotype in TCTP-overexpressing transgenic mice [40,41]. Clearly, all these interactions involving TCTP are important and still need to be better understood and explained. Our laboratory has long been interested in the interaction of TCTP with Na,K-ATPase and has made some important contributions to the topic. This review covers only the interaction of TCTP with Na,K-ATPase, summarizing what is currently known on the interaction and its biological implications.

## 3. Na,K-ATPase–TCTP Interaction in Molecular Terms

TCTP acts as a cytoplasmic repressor of Na,K-ATPase by interacting with its third cytoplasmic domain. TCTP interaction with Na,K-ATPase α1 and α2 subunits was first discovered using the yeast-two hybrid system [45] and then confirmed by co-immunoprecipitation assays in HeLa [49] and MCF10A cells [29]. The third cytoplasmic domain of Na,K-ATPase α subunit was fused to the LexA DNA-binding domain and was used to isolate several cDNA clones from a rat skeletal muscle library, one of which was later identified as TCTP. Using yeast and mammalian cells, the C-terminal region of TCTP was discovered to be essential for the interaction with and inhibition of Na,K-ATPase. TCTP binding to Na,K-ATPase inhibited its pumping activity without decreasing its mRNA and protein level [45].

The conformational changes in Na,K-ATPase α subunit resulting from the interaction with TCTP affected the binding of PI3K and Src to Na,K-ATPase [29]. PI3K is known to interact with the N-terminal proline-rich motif of Na,K-ATPase α subunit [50], and PI3K p85 protein is released and activated after TCTP overexpression [29]. It is reported that the SH3–SH2 domain of Src interacts with the second cytoplasmic domain of Na,K-ATPase α1, and the kinase domain of Src interacts with the third cytoplasmic domain of Na,K-ATPase α1. After ouabain treatment, the SH3–SH2 domain of Src remains bound to Na,K-ATPase α1, and only the kinase domain of Src is exposed to induce signaling pathways [51]. Unlike the ouabain-induced Na,K-ATPase signaling pathway which only exposes Src kinase domain [51], TCTP overexpression releases Src from Na,K-ATPase and phosphorylates Src Tyr 418 residue in human breast epithelial cell, thus activating a Na,K-ATPase-induced tumorigenic signaling pathway [29].

## 4. Diverse Biological and Pathological Effects of Na,K-ATPase–TCTP Interaction

### 4.1. Na,K-ATPase Inhibition by TCTP Is Implicated in Hypertension and Cataracts in Mice

**Figure 1:**
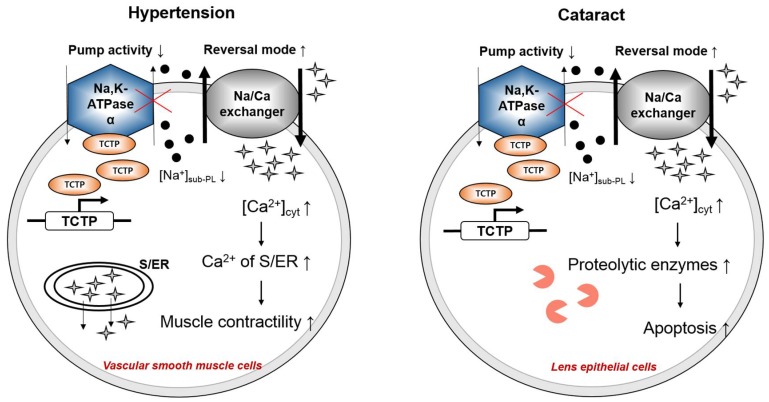
Mechanisms behind hypertension and cataract in TCTP-overexpressing transgenic mice. Systemic arterial hypertension and cataract are induced through the inhibition of Na,K-ATPase, which leads to accumulation of Na^+^ and increase in cytoplasmic Ca^2+^ mobilization. Increased Ca^2+^ levels in sarcoplasmic reticulum and endoplasmic reticulum(S/ER) cause an increase in vascular smooth muscle contractility, inducing hypertension. In lens epithelial cells, the increased Ca^2+^ mobilization activates proteolytic enzymes that cause apoptosis and cataract. (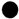, Na^+^; 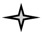 Ca^2+^; 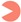, proteolytic enzymes).

In order to investigate the biological effects of Na,K-ATPase–TCTP interaction, Kim et al. [40,41] generated TCTP-overexpressing transgenic mice with different backgrounds using the chicken β-actin promoter: one type of transgenics in the C57BL/6 background and another type in the C57BL/6 + CBA hybrid background which were backcrossed with C57BL/6 mice. TCTP-induced Na,K-ATPase inhibition in both types of TCTP-overexpressing transgenic mice led to systemic arterial hypertension as early as six weeks after birth, with no significant cardiac dysfunction (Figure 1) [40,41]. Na,K-ATPase is a key molecule that controls cellular ion homeostasis [8] and regulates smooth muscle contractility in the vasculature [52,53]. The inhibition of Na,K-ATPase by ouabain [54] and by endogenous ouabain-like compounds [55] is implicated in the development of hypertension. Likewise, the vascular smooth muscle cells of TCTP-overexpressing transgenic mice showed increased sensitivity to vasoconstrictors such as norepinephrine and serotonin, increased Ca^2+^ levels at resting state, and increased intracellular calcium mobilization, which all ultimately led to an augmented contractile response to vasoconstrictors and an attenuated relaxation response to vasodilators (Figure 1) [40]. One of the explanations for the increased Ca^2+^ levels following TCTP inhibition of Na,K-ATPase invoked the involvement of a Na^+^/Ca^2+^ exchanger, which works in reversal mode, pumping out Na^+^ and pumping in Ca^2+^ [56]. Apolipoprotein E is an important glycoprotein that acts as a ligand for chylomicron-remnant receptor, and lack of apolipoprotein E is known to induce atherosclerosis in mice [57]. TCTP overexpression in apolipoprotein E knockout mice exacerbated the atherosclerotic lesions, suggesting that the overexpression of TCTP accelerates the development of atherosclerotic lesions caused by a high-fat diet without significantly altering plasma lipid profiles [58].

Na,K-ATPase is critical for the regulation of Na^+^ and K^+^ levels in the lens [59]. In human age-related cortical cataract lens as well as in diabetic cataract lens, Na^+^ levels are abnormally elevated, while K^+^ levels are abnormally lowered, suggesting a disturbed ion and fluid homeostasis. This disturbance is known to decrease the transparency of the lens causing cataractogenesis [60,61]. Also, fluid accumulation and cellular or protein damage from the activation of calcium-dependent proteases are reported to induce opacification of the lens [62]. Since TCTP, as a cytoplasmic repressor of Na,K-ATPase [45], is known to increase intracellular calcium levels [40], Kim et al. investigated whether opacification of the lens is common in TCTP-overexpressing transgenic C57BL/6 N mice and confirmed that TCTP inhibition of Na,K-ATPase led to a higher incidence (7.38%) of cataracts in TCTP transgenic mice than in the controls (1.47%) (Figure 1). A transient overexpression of TCTP inhibited the pumping activity of Na,K-ATPase without affecting its expression level and caused increased Ca^2+^ mobilization in lens epithelial cells [41]. It is reported that intracellular Ca^2+^ accumulation induces the activation of proteolytic enzymes, loss of cellular integrity, and apoptosis of cells, which lead to lens opacification [63]. The incidence rate of cataract formation was increased only in transgenic C57BL/6 N mice but not in transgenic C57BL/6 + CBA hybrid mice, which acquired systolic hypertension but showed resistance to cataract formation for yet unknown reasons. However, since the incidence of hypertension is known to be associated with cataract formation in humans [64], the pathological phenotype of TCTP-overexpressing transgenic mice, hypertension, and cataractogenesis may occur as a consequence of the pathologic effects of Na,K-ATPase inhibition.

### 4.2. Role of Na,K-ATPase–TCTP Interaction in Tumorigenesis

**Figure 2:**
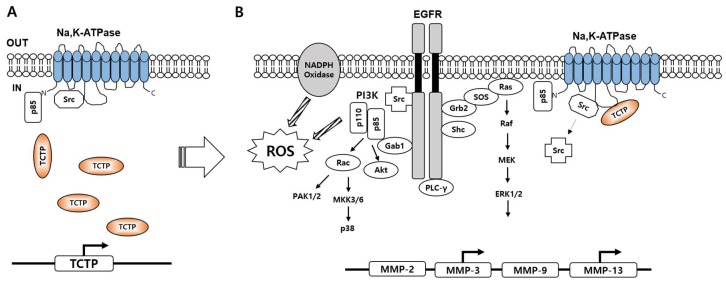
A schematic overview of TCTP-induced Na,K-ATPase signaling pathways. (**A**) PI3K p85 subunit and Src are constitutively bound to Na,K-ATPase α subunit in the normal state; (**B**) When TCTP is overexpressed, TCTP interacts with Na,K-ATPase α subunit and induces Na,K-ATPase conformational changes that result in Src and p85 release. Activated Src transactivates PI3K–AKT, Ras–Raf–MEK–ERK1/2, Rac–PAK1/2, MKK3/6–p38 and phospholipase C (PLC)-γ signaling pathways. TCTP enhances NADPH oxidase-dependent reactive oxygen species (ROS) and induces matrix metalloproteinase (MMP)-3 and -13.

TCTP is involved in tumorigenesis [65,66], and various studies demonstrated its anti-apoptotic activity [67]. Na,K-ATPase is known not only for its pumping activity, but also for its signaling activity [68]. TCTP-induced Na,K-ATPase inhibition activates the signal transducing role of Na,K-ATPase (Figure 2) [29]. Kim et al. demonstrated that TCTP overexpression in HeLa cells inhibits the pumping activity of Na,K-ATPase and induces the phosphorylation of EGFR at Tyr 845, 992, 1068, and 1148 [49]. The key molecule that induces EGFR activation after TCTP overexpression was discovered to be Src. Src kinase directly phosphorylates EGFR at Tyr 845 [69] and promotes cell adhesion, motility, and cancer development [70]. In TCTP-overexpressing MCF10A cells, TCTP interaction with α1 subunit of Na,K-ATPase released and activated Src [29]. EGFR was not activated in TCTP-overexpressing Src-deficient mouse embryonic fibroblasts (SYF), and addition of Src caused the phosphorylation of EGFR at Tyr 845. Activated Src induced EGFR phosphorylation at Tyr 845, 992, 1086, 1148, and 1173 in MCF10A cells. The differences in the phosphorylation of the tyrosine residues of EGFR of HeLa and MCF10A cells may due to differences in the cell lines, but tyrosine 845 of EGFR was activated in both cell lines, suggesting the importance of Src activation in TCTP-induced EGFR transactivation and stimulation of cancer-related signaling pathways.

Different signaling pathways of EGFR are mediated through binding of adaptor proteins [71]. Grb2 is located in the cytosol, constitutively bound to the Ras exchange factor Sos. Phosphorylation at EGFR Tyr 1068 and 1086 recruits Grb2 and Shc adaptor molecules [72] and activates Ras, by exchanging Ras-bound guanosine diphosphate (GDP) to guanosine triphosphate (GTP) [73], and the Ras–Raf–ERK signaling pathway. Likewise, TCTP-induced EGFR activation increases the binding of Grb2, Gab1, and Shc adaptor proteins to EGFR and activates Ras-dependent downstream signaling molecules such as Raf, MEK, and ERK1/2 in TCTP-overexpressing MCF10A cells [29]. In TCTP-overexpressing HeLa cells, Ras activity is increased, and ERK1/2 are activated; the activation of both pathways is diminished after treatment with the tyrosine kinase inhibitor genistein and the MEK inhibitor PD98059 [50], suggesting that the Ras–Raf–MEK–ERK1/2 pathway functions downstream of TCTP-induced EGFR transactivation.

Along with the Ras–Raf–MEK–Erk1/2 cascade, the Rac–MKK3/6–p38, PAK1/2 and Rac–PI3K–AKT pathways are activated upon TCTP overexpression [29]. MKK3/6 and p38 phosphorylation was lower compared to Rac activation, which increases markedly in TCTP-overexpressing MCF10A cells. p21-activated kinases (PAK) are serine/threonine kinases that induce cytoskeletal remodeling, cell motility, cell proliferation, and mitotic abnormalities, all of which promote tumor formation [74]. Parallel with Rac activation, PAK1/2 are also activated in TCTP-overexpressing MCF10A cells. The Rac–PI3K–AKT pathway in MCF10A and HeLa cells was also activated after TCTP overexpression. The anti-apoptotic effect of TCTP was dependent on the activation of AKT, which was abrogated by the PI3K inhibitor LY294002 [50]. In addition, TCTP overexpression stimulated reactive oxygen species (ROS) production by NADPH oxidase through Src and PI3K activation [29].

### 4.3. Role of Na,K-ATPase–TCTP Interaction in Cell Migration and Matrix Metalloproteinase (MMP) Upregulation

TCTP-induced phosphorylation of EGFR at Tyr 992 activated PLC-γ [29], which is a key molecular switch that regulates tumor migration [75]. PP2, LY29002, and U73122, which are a Src inhibitor, PI3K inhibitor, and PLC-γ inhibitor, respectively, blocked cell motility in TCTP-overexpressing MCF10A cells, indicating the importance of Src, PI3K. and PLC-γ activation in TCTP-induced cell migration. Also, TCTP-induced cell migration in HeLa cells was blocked by LY294002, a PI3K inhibitor, but not by U0126, a MEK inhibitor, demonstrating that TCTP-induced cell migration is dependent on the PLC-γ pathway, but not on the MAPK pathway [50]. The above findings underscore the importance of the PLC-γ pathway in TCTP-induced cell migration.

Matrix metalloproteinases (MMPs) are endoproteinases that function in extracellular matrix degradation [76]. Abnormal MMP expression promotes tumor growth, invasion, and metastasis [77]. TCTP-overexpressing MCF10A cells showed upregulated MMP-3 and MMP-13 [29], and both MMPs serve as tumor promoters [78,79]. In TCTP-upregulated LLC-PK1 cells, MMP-9, which is known to be involved in cell invasion, was increased [31]. Since the MAPK signaling pathway activates AP-1 proteins, such as c-jun and c-fos, which promote transcription of MMPs [80], TCTP overexpression likely promotes the expression of MMPs through the activation of the MAPK signaling pathway. The difference in the subtypes of MMPs involved in invasion may be due to differences in the cell lines; however, the results underscore MMPs involvement in TCTP-induced cell invasion.

### 4.4. Other Biological Phenomenon in Which Na,K-ATPase Inhibition by TCTP May Possibly Play Role

Macroautophagy (also referred to as autophagy) is a catabolic process in which proteins and organelles are engulfed by a membrane structure called phagophore, which elongates to form a double-membrane vesicle called autophagosome. Autophagy is involved in a variety of cellular functions, including the nutrient sensing response, cell growth, homeostasis, and cell death [81]. Autophagy is a dynamic process that can be divided into three consecutive processes: formation of autophagosomes, generation of autolysosome, and degradation [82]. Class III PI3K activates AKT and induces mTOR activation that inhibits autophagy [83], and TCTP is known to induce the activation of PI3K by inhibition of Na,K-ATPase [29]. Thus, TCTP involvement in autophagy was investigated by our research group, and it was discovered that TCTP negatively regulates autophagy in vitro and in vivo [84].

#### 4.4.1. TCTP Downregulation Interrupts BECN1 Interactome

In HeLa cells, TCTP was reported to interact with Na,K-ATPase and inhibit its pumping activity [46]; also, TCTP inhibition using TCTP siRNA caused activation of Na,K-ATPase, suggesting that endogenous TCTP inhibits Na,K-ATPase activity [85]. Since some reports were published describing how Na,K-ATPase inhibition by cardiac glycosides induces autophagy in various cells [86], our group investigated whether TCTP, a cytoplasmic Na,K-ATPase inhibitor, regulates autophagy. In TCTP-silenced HeLa cells and mouse embryonic fibroblast cells from TCTP heterozygote knockout mice (TCTP^+/−^), the number of GFP-LC3 puncta and lipid-bound GFP-LC3-II level increased, while SQSTM1 level decreased [84]. LC3B-I is converted to LC3B-II by Atg3, and LC3B-II is located on both internal and external surfaces of the autophagosome, helping to select cargoes for degradation [82]. Also, SQSTM1 is an ubiquitin-binding scaffold protein that binds directly to LC3 and recruits protein aggregates to the autophagosome, which are then degraded by autophagic flux [87]. Thus, the increase in the number of GFP-LC3 puncta and the decrease in SQSTM1 level indicated autophagy induction [88], which could be observed in TCTP downregulated cells.

The interaction of BECN1 and class III PI3K induces vesicle nucleation in autophagy; BCL2 is an anti-apoptotic protein that binds to BECN1 and inhibits the interaction between BECN1 and class III PI3K, ultimately repressing autophagy [89]. TCTP-downregulated cells showed decreased BCL2 expression caused by increasing mitogen-activated protein kinase 8 (MAPK8) expression and phosphorylation [84]. MAPK8–JNK1 phosphorylates BCL2, which leads to BCL2 degradation during starvation, resulting in decreased interaction between BECN1 and BCL2 [90]. Since BECN1 expression level of TCTP-downregulated cells did not change, TCTP affects BCL2 by regulating MAPK8–JNK1 activity. The interaction of the catalytic subunit of PI3K and UV radiation resistance-associated gene protein (UVRAG) with BECN1 were significantly increased in TCTP knockdown HeLa cells [84]. BECN1 interaction with class III PI3K initiated vesicle nucleation, and UVRAG addition induced autophagosome formation and maturation [91]. Thus a reduction in TCTP expression stimulates both autophagosome formation and maturation by regulating BECN1 interactome; in other words, TCTP knockdown stimulates the on-rate of autophagy.

#### 4.4.2. TCTP Inhibition Induces Cellular Autophagy via the mTORC1 and AMPK Pathway

TCTP downregulation induces cellular autophagy via mTORC1 and adenosine monophosphate-activated protein kinase (AMPK) pathways. It has been reported that TCTP activates mTORC1 pathway by promoting the GDP–GTP exchange of Rheb [25,92]. In TCTP-downregulated HeLa cells, the phosphorylation levels of ribosomal protein S6 kinase (p70S6 kinase), eukaryotic initiation factor 4E-binding protein 1 (4E-BP1), and ULK1 Ser757, which are all mTORC1 downstream signaling molecules, are decreased, and the total expression level of mTOR is also reduced [84]. Rapamycin induces autophagy by acting as an allosteric inhibitor of mTORC1 [93]. In accordance with the report describing partial rapamycin resistance in the 4E-BP1–eIF4E effector pathway [94], rapamycin treatment alone only partially inhibited 4E-BP1 and p70S6 kinase in HeLa cells [84]. TCTP knockdown synergized with rapamycin-induced autophagy by further dephosphorylating p70S6 kinase and 4E-BP1 and reducing total mTOR expression, suggesting that TCTP-mediated inhibition of autophagy occurs in part through a rapamycin-independent pathway [84]. Furthermore, TCTP downregulation induced autophagy by phosphorylating AMPKα Thr172 under normoxic conditions [95]. TCTP downregulation activates AMPK, which induces phosphorylation of acetyl-CoA carboxylase, a downstream molecule of the AMPK pathway [84]. In contrast, TCTP knockdown did not potentiate starvation-induced autophagy [84] and, rather, inhibited autophagy in starvation or hypoxic condition by inhibiting the phosphorylation of AMPKα Thr172 [95]. Thus, TCTP regulates the mTORC1 and AMPK pathwas in various conditions, functioning as an autophagy regulator.

**Figure 3:**
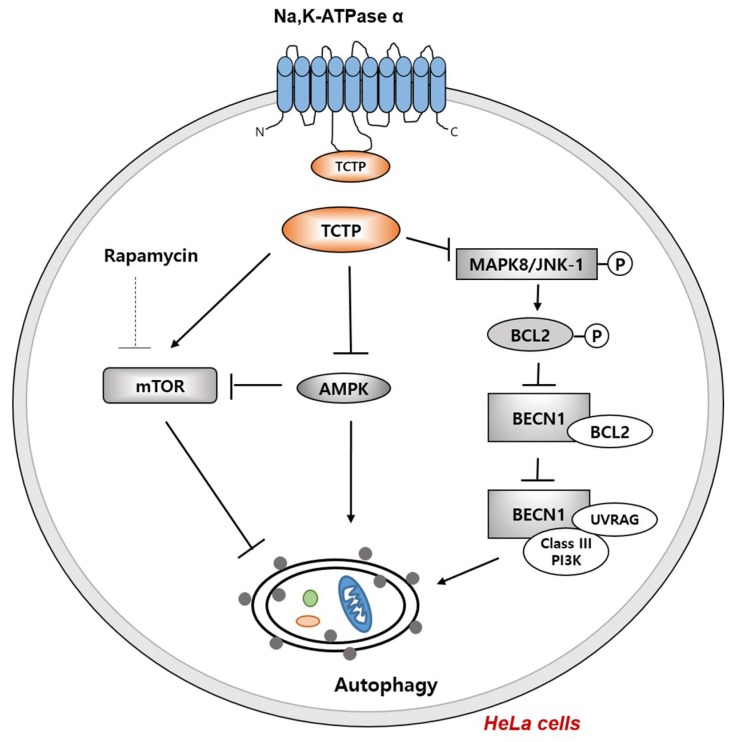
TCTP as a negative regulator of autophagy. TCTP negatively regulates AMPK, which leads to mTORC1 activation that promotes cell proliferation and inhibits autophagy in HeLa cells. TCTP also inhibits MAPK8–JNK1 which is known to phosphorylate and degrade BCL2. BCL2 forms a complex with BECN1 in the normal state, inhibiting autophagy. Once BCL2 is phosphorylated and degraded upon stress or starvation, BECN1 forms a complex with class III PI3K and UV radiation resistance-associated gene (UVRAG) and induces autophagy.

TCTP functions to inhibit autophagy by controlling mTORC1-independent pathways like MAPK8–BCL2–BECN1, whereas downregulation of TCTP enhances rapamycin-induced autophagy through the inhibition of the mTORC1 pathway and the activation of the AMPK pathway (Figure 3). Thus, TCTP knockdown induces autophagy through both mTORC1-dependent and mTORC1-independent pathways.

## 5. Concluding Remarks

This review attempts to explain the manifold functions of TCTP and Na,K-ATPase interaction in various biological phenomena, including tumorigenesis. Several studies have demonstrated that TCTP inhibits Na,K-ATPase by binding to its third cytoplasmic domain and induces pathophysiological events such as hypertension and cataractogenesis in TCTP-overexpressing transgenic mice. The novel function of TCTP in hypertension has been explained as due to its interaction with and inhibition of Na,K-ATPase. TCTP has been implicated in cancer development in numerous researches, but its exact role in tumor progression has not been understood until the discovery of its interaction with Na,K-ATPase. Thus, the hypertensive, cancer-related, and other functions of Na,K-ATPase–TCTP interaction reviewed here suggest that TCTP interaction with Na,K-ATPase might represent a potential therapeutic target in selected diseases.

## Figures and Tables

**Table 1:** Translationally controlled tumor protein (TCTP) interacting partners.

Interacting Partners	Array	Significance	Reference
Bcl-xL	IP, Pull-down	The N-terminal region of TCTP interacts with Bcl-xL and increases stability of Bcl-xL	[34]
eEF1B	Crystallography, MS	TCTP interacts with the central acidic region of eEF1B	[42]
Hsp27	Yeast two-hybrid, IP	Hsp27 interacts with TCTP and protects TCTP from ubiquitination	[43]
Mcl-1	Yeast two-hybrid, Pull-down, IP	Interaction between TCTP and Mcl-1 increases the stability of the two proteins	[35,44]
MDM2	Pull-down, IP	TCTP interacts with the N-terminal region of MDM2 and inhibits ubiquitination of MDM2	[39]
Na,K-ATPase	Yeast two-hybrid, Pull-down, IP	TCTP interacts with the third cytoplasmid domain of Na,K-ATPase α subunit and inhibits its pumping activity. TCTP induces Na,K-ATPase-mediated tumorigenic signaling pathways	[29,45]
p53	IP, Pull-down	TCTP forms a complex with p53 and MDM2 and promotes the degradation of p53	[38]
Plk	Pull-down, IP	Plk interacts and phosphorylates TCTP, inhibiting the microtubule-stabilizing activity of TCTP	[26]
Tubulin	IP, Pull-down	TCTP interacts with tubulin during most of the cell cycle phases (G1, S, G2, and early M phase) and not during the resting state	[34,46]
VHL	MS, IP	TCTP interacts with VHL and promotes ubiquitination of VHL, leading to its degradation	[47,48]

Arrays: IP, immunoprecipitation; MS, mass spectrometry; eEF1B, eukaryotic elongation factor 1B; Hsp27, heat shock protein 27; Mcl-1, myeloid cell leukemia 1 protein; MDM2, mouse double-minute 2 homolog; Plk, polo-like kinase; VHL, von Hippel–Lindau protein.

## Abbreviations

4E-BP1	eukaryotic initiation factor 4E-binding protein 1
AMPK	adenosine monophosphate-activated protein kinase
CG	cardiac glycoside
CRPC	castration-resistance prostate cancer
eEF1B	elongation factor 1 B
EGFR	epidermal growth factor receptor
EMT	epithelial to mesenchymal transition
Hsp27	heat shock protein 27
MAPK8	mitogen-activated protein kinase 8
Mcl-1	myeloid cell leukemia 1 protein
MDM2	mouse double-minute 2 homolog
MMP	matrix metalloproteinase
p70S6	ribosomal protein S6 kinase
PAK	p21-activated kinase
PLC-γ	phospholipase-γ
Plk	polo-like kinase
PI3K	phosphatidylinositide 3-kinase
ROS	reactive oxygen species
TCTP	translationally controlled tumor protein
TM	transmembrane
UVRAG	UV radiation resistance-associated gene
VHL	von Hippel–Lindau protein
